# CAR-T Cell Therapy in Cancer: Tribulations and Road Ahead

**DOI:** 10.1155/2020/1924379

**Published:** 2020-01-17

**Authors:** Qingyang Zhang, Jieming Ping, Zirui Huang, Xiaoli Zhang, Jingyi Zhou, Gangyang Wang, Shaoyang Liu, Jianjun Ma

**Affiliations:** ^1^Human Anatomy Laboratory, School of Basic Medicine, Xinxiang Medical University, Henan 453003, China; ^2^Division of Histology and Embryology, School of Basic Medical Sciences, Tongji Medical College, Huazhong University of Science and Technology, Wuhan 430030, China; ^3^Human Anatomy Laboratory, School of International Education, Xinxiang Medical University, Henan 453003, China; ^4^Department of Orthopaedics, Shanghai Bone Tumor Institute, Shanghai General Hospital, Shanghai Jiao Tong University School of Medicine, Shanghai 200080, China; ^5^Department of Orthopedics, Shanghai Putuo District Central Hospital, Shanghai 200062, China

## Abstract

Chimeric antigen receptor- (CAR-) T cell therapy is one of the most recent innovative immunotherapies and is rapidly evolving. Like other technologies, CAR-T cell therapy has undergone a long development process, and persistent explorations of the actions of the intracellular signaling domain and make several improvements have led to the superior efficacy when anti-CD19 CAR-T cell treatments in B cell cancers. At present, CAR-T cell therapy is developing rapidly, and many clinical trials have been established on a global scale, which has great commercial potential. This review mainly describes the toxicity of CAR-T cell therapy and the challenges of CAR-T cells in the treatment of solid tumors, and looks forward to future development and opportunities for immunotherapy and reviews major breakthroughs in CAR-T cell therapy.

## 1. Introduction

Although cancer treatments have undergone massive developments in recent year, cancer remains a difficult disease to solve worldwide. Traditional cancer therapies, such as clinical operation, chemotherapy, and radiotherapy, may have a curative effect in the short term but will cause side effects, decreasing cancer patient quality of life [1]. Immunotherapy has been recognized as a new generation of an antitumor weapons and will be the leading force in future cancer treatment. Immunotherapy is a kind of therapy that targets the human immune system rather than directly targeting tumors. It can resist and kill tumor cells by activating patient defenses [2]. Adoptive cell transfer therapy (ACT) is an immunotherapy that separates immunocompetent cells from cancer patients and transfers them to patients after expansion or functional identification in vitro; adoptive cells kill tumor cells directly or stimulate the body's immune response [3].

ACT can be roughly divided into three forms. (1) Tumor-infiltrating lymphocytes (TILs) are lymphocytes that infiltrate the tumor cell stroma, and after IL-2 activation, they have a stronger antitumor effect. While melanoma patients showed a remarkable clinical response by TILs, TIL treatment was not as effective in other tumors, such as renal cell carcinoma [4, 5]. (2) T cell receptor- (TCR-) T cells are heterodimeric proteins composed of two structural domains: TCR*α* and TCR*β*. TCR-T cells activate cytotoxicity and release cytokines to inhibit the proliferation of cancer cells specifically or kill cancer cells by explicitly recognizing the assembly, modification and processing of specific proteins in cancer cells via cancer-specific major histocompatibility complex (MHC) molecules [6, 7]. TCR-T cells can target most tumor-specific antigens, particularly those that can recognize the tumor cell antigen. Thus, TCR-T cells can recognize a broader range of antigens than the tumor antibody drugs [4, 8]. (3) Chimeric antigen receptor- (CAR-) T cells are composed of extracellular, transmembrane, and intracellular domains. The extracellular domain has an scFv domain for the recognition of tumor-associated antigens with specificity and affinity. The intracellular domain is derived from the immunoreceptor tyrosine-based activation motif (ITAM) of the TCR complex CD3*ζ* chain, which activates the costimulatory signal. CAR-T cells are manufactured by generating a single-chain variable fragment (scFv) that recognizes tumor-associated antigen (TAA) recombinants and an intracellular, recombinant “immunoreceptor tyrosine activation motif” (ITAM) region, which are incorporated into a recombinant plasmids in vitro. Subsequently, the recombinant plasmid is transduced into T cells, allowing T cells to express the appropriate tumor surface antigen receptors, and T cells are expanded after transfection. CAR-T cells recognize and kill tumor cells independent of major histocompatibility complex (MHC) molecules; thus, immune escape of tumor cells overcome by the decreased expression of MHC molecules. However, CAR-T cells can recognize tumor antigens only when they are specifically expressed on the surface of cell membranes; thus, the target is very specific [9].

**Figure 1:**
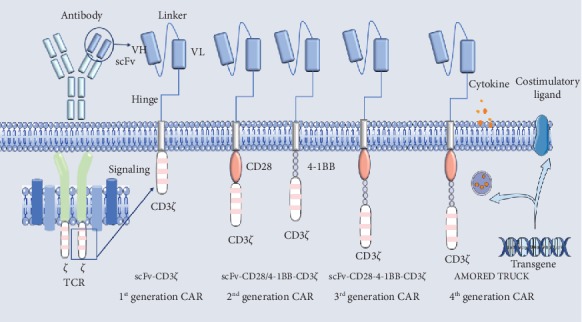
The development and design principle of CAR-T three generations. The first generation of CAR-T cells was composed of immunoglobulin scFv and CD3 complexes. Most of the experiments did not respond well in cell expansion, in vivo survival time, cytokine secretion, etc., and the therapeutic effect was not as expected. The second- and third-generation CARs add costimulatory molecules such as CD28, CD134, and CD137 (4-1BB) to the chimeric receptor, which enables the cells to obtain long-lasting in vitro proliferation ability and strong cytokine secretion ability. The fourth generation of CAR-T can solve the problem that traditional CAR-T cannot identify and remove some antigens that are not explicitly recognized by T cells.

To develop the best CAR-T cells, four generations of CAR-T cells have been created via continuous exploration and improvement of the effects of intracellular signaling domains (Figure 1). The first generation of CAR-T cells includes an scFv antigen-binding epitope with one signaling domain. The CD3*ζ* chain activates the first generation of CAR-T cells. The CD3*ζ* chain provides the signals required for T cell activation, lysis of target cells, regulation of IL-2 secretion, and antitumor immunoregulatory activity. However, the antitumor action of the first-generation CAR-T cells was limited in vivo, and the decrease in T cell proliferation ultimately led to the apoptosis of T cells [10, 11]. The second-generation CAR-T cells add an additional costimulatory signal to the cells. The commonly used costimulatory molecule is CD28 or the 4-1BB receptor (CD137). Many studies have shown that the second-generation CAR-T cells have no specific antigen, and compared with the those of first-generation CAR-T cells, second-generation CAR-T cell proliferation, cytokine secretion, and secretion of antiapoptotic proteins are increased, and the second-generation cells lead to delayed antigen-induced cell death [8]. To further improve the design of CAR-T cells, many research groups began to focus on the development of third-generation CAR-T cells. Wilkie et al. showed that there was no significant difference in antitumor cytotoxicity between second-generation CAR-T cells and third-generation CAR-T cells incorporating the 4-1BB and CD28 signaling domains, although T cells expressing the third-generation CAR-T cells were able to secrete larger amounts of IFN-*γ* than those with first-generation or second-generation CAR-T cell [12]. Some studies have shown that CD28 exhibits improved antitumor activity, and the advantage of 4-1BB is to prolong the survival of T cells and maintain their anticancer effects. However, recent results show that only the second-generation CAR-T cells can activate CD3*ζ*, and second-generation CAR-T cells have stronger signal transduction and antitumor effects than third-generation CAR-T cells [13]. Fourth-generation CAR-T cells are also known as T cells redirected for universal cytokine killing (TRUCK). Unlike earlier generation of CAR-T cells, these CAR-T cells can identify and remove some antigens that are not recognized explicitly by T cells. The fourth-generation CAR-T cells contain an activated T cell nuclear factor transcriptional counterpart that allows them to secrete specific cytokines (e.g., IL-12) in the tumor, thereby modifying the tumor microenvironment and recruiting and activating other the immune cells to generate an immune response.

## 2. Potential Mechanisms of CAR-T Cell-Mediated Toxicity

**Figure 2:**
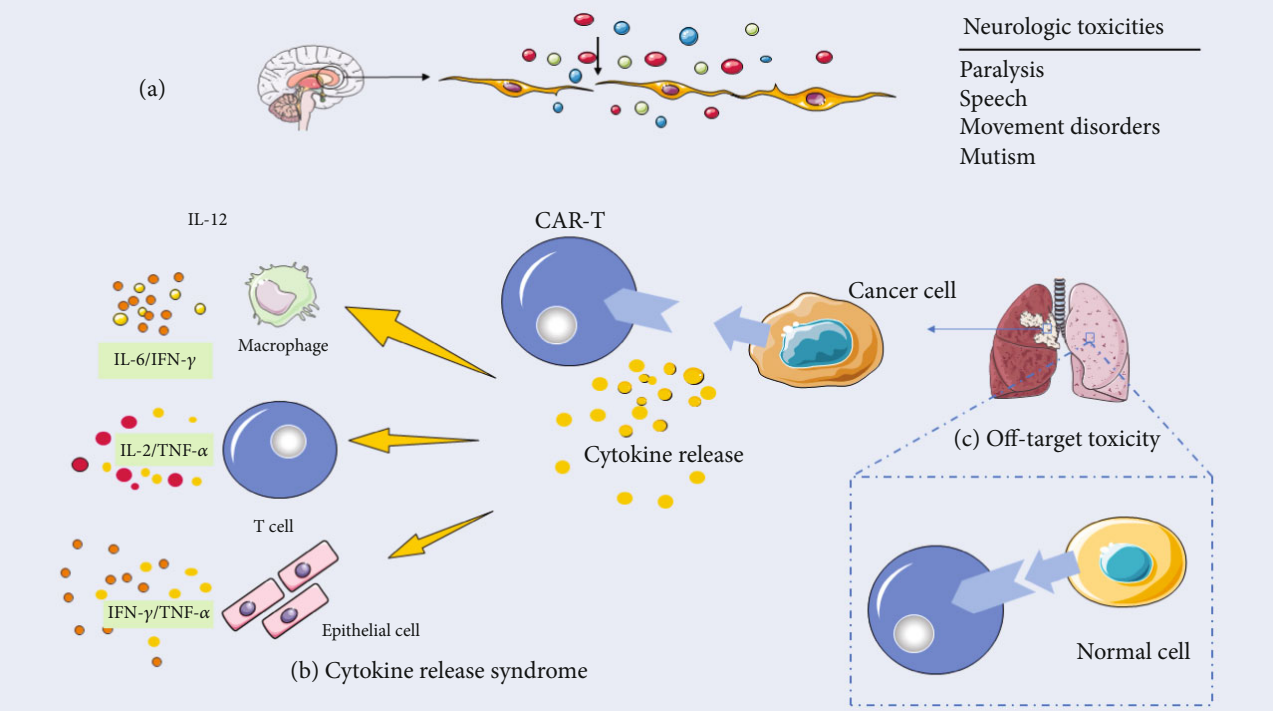
Common side effects of CAR-T cell therapy. (a) CAR-T cells can be examined in routine examinations of cerebrospinal fluid, which allows increased transport of CAR-T cells and other lymphocytes to the central nervous system and increases permeability to soluble mediators. (b) Cytokine storms are the most common form of CAR-T toxicity. The affinity and conduction function of CAR-T cells cause rapid release of a large number of cytokines such as TNF-*α*, IL-1, IL-2, IL-6, IFN-*α*, and IFN-*γ* to cause acute respiratory distress syndrome after binding to relevant antigens and multiple organ failure. (c) Off-target effects are the effect of cells on additional targets outside of the design, leading to autoimmune disease responses to normal tissues.

Significant progress has been made in the field of cancer immunotherapy, and CAR-T cells have shown outstanding efficacy in clinical trials. As with all technologies, CAR-T technologies also need to go through a long process of development, and CAR-T cell therapy has related acute and chronic toxicities that have become a roadblock on the developmental path (Figure 2). If these setbacks are not overcome, it will be difficult to make a more significant breakthrough. However, these barriers also represent opportunities in this field [14].

### 2.1. Cytokine Release Syndrome

Cytokine release syndrome (CRS) is the most common toxic side effect in CAR-T cell therapy [15]. CRS is a systemic inflammatory response caused by the significant increase in cytokines accompanied by the rapid in vivo activation and proliferation of CAR-T cells, usually occurring within a few days after the first infusion [3, 16]. CRS is a clinical condition with mild symptoms of fever, fatigue, headache, rash, joint pain, and myalgia. Severe CRS cases are characterized by tachycardia, hypotension, and high fever [17, 18]. Mild to moderate CRS is usually self-limiting and can be managed through close observation and supportive care. Severe CRS must be treated with tocilizumab or steroids alone for intensive treatment. However, there are still cases in which the clinical symptoms are not improved or aggravated after intensive treatment [19]. CRS is caused by excessive inflammatory cytokines as a result of hyperimmune activation. Indeed, the detailed pathogenesis is not yet clear. However, recent discoveries have revealed the underlying mechanisms of CAR-T cell-induced CRS. After the scFv of CAR-T cells contacts the target antigen, CAR-T cells proliferate, become activated, and secrete a large number of cytokines such as IL-6, IL-10, TNF-*α*, and IFN-*γ* in a short time [20, 21]. The released cytokines activate immune cells (e.g., macrophages and T cells) as well as nonimmune cells (e.g., epithelial cells) to release more cell types and quantities of cytokines [22, 23]. Studies have shown that IFN-*γ* activates macrophages and induces the release of TNF-*α*, IL-6, IL-15, IL-1*β*, and IL-12, maintaining or enhancing the subsequent immune responses [24]. Among these cytokines, IL-6 is one of the key cytokines. CAR-T cell-related IL-6 is mainly produced by tumor-specific macrophages and depends on direct contact between CAR-T cells and macrophages. The key signal is mediated by CD40/CD40L on the cell surface. The level of IL-6 produced by macrophages correlates with the expression level of CD40L on the surface of CAR-T cells [25]. Clinically, tocilizumab, siltuximab, JAK kinase inhibitors, and corticosteroids blocking IL-6 can rapidly reverse fever, hypotension, and hypoxia [3]. Two other studies have shown that severe CRS is associated with the activation or dysfunction of endothelial cells [26]. Available data indicate that VWF and Ang-2 elevate the levels of biomarkers of endothelial activation in severe CRS patients receiving CAR-T cell treatment [27]. The results of this study can help explain the mechanism by which tocilizumab alleviates CRS.

### 2.2. Neurologic Toxicity

CAR-T cell treatment of leukemia causes neurological symptoms, which is an unexpected and unclear phenomenon. Neurologic toxicity was very common in the CD19-specific CAR-T cell trial, but its pathogenesis was not very clear [28–30]. Several research groups have reported that these symptoms are diverse but minor symptoms can resolve on their own, such as paralysis, speech disorders, movement disorders, autism, and seizures [31, 32]. However, this unexpected toxicity can also cause death [33]. Previous studies showed that intravenous CAR-T cells were observed in cerebrospinal fluid, demonstrating that CAR-T cells can pass the blood-brain barrier, and that these CAR-T cells can be transported to the central nervous system to treat malignant nervous system tumors [28, 34, 35]. There is also evidence that the neurotoxicity may be caused by inflammatory cytokines leading to endothelial damage and inducing CAR-T cell-related encephalopathy syndrome (CRES) [31, 36]. Therefore, elevated cytokines may affect the central nervous system, and CAR-T cells may also affect brain tissue directly, causing a series of neurological side effects [37]. In addition, clinical studies have shown that the occurrence of neurologic toxicity is related to the premature peak concentrations of cytokines such as TNF-*α*, iIL-6, and IFN-*γ*. High concentrations of secreted cytokines activate endothelial cells, resulting in increased levels of Ang-2 and VWF and leading to increased permeability of the blood-brain barrier [38, 39]. However, the mechanisms of these symptoms remain to be confirmed. Tocilizumab cannot effectively solve the problem of neurologic toxicity caused by CAR-T cells. Tocilizumab cannot cross the blood-brain barrier, but the CNS permeability of CAR-T cells is strong [40]. Therefore, corticosteroids have become the first-line treatment in some institutions for neurologic toxicity.

### 2.3. Off-Tumor Toxicity

After genetic modification, antigen receptors on the surface of T cells target tumor cells by identifying specific antigens on the surface of tumor cells [36]. However, these particular antigens may also be present in normal tissue cells, so the injection of CAR-T cells may damage normal tissues and organs, which is called the on-target/off-target tumor effect [41, 42].

The TAA recognized by CAR-T cells is not usually unique to tumor cells, and when CAR-T cells contact nontumor target antigens, it causes an off-target phenomenon. To avoid the off-target effect, double-antigen-reporting CAR-T cells have emerged. First, CAR-T cells recognize tumor cell antigen A and activate the expression of intracellular CAR-encoding sequences. After CAR expression, the surface single-chain antibody recognizes antigen B, thereby preventing CAR-T cells from killing tumor cells.

## 3. Advances in Research of CAR-T Cell Therapy for Solid Tumors

CAR-T cells have had a remarkable success in the treatment of hematological tumors, but there are some difficulties still in the treatment of solid tumors. When CAR-T cells are used to treat solid tumors, we face three main challenges: (1) lack of proper targets and heterogeneity, (2) CAR-T cells are not effectively infiltrating into tumor tissue, and (3) effect of tumor microenvironment on CAR-T cell therapy [43].  Table 1 lists several difficulties and solutions for the application of CAR-T cell therapy to solid tumors. Although early CAR-T cell trials of solid tumors did not show the same success as observed in leukemia trials, a better understanding of the multiple barriers seen in solid tumors could promote the design of clinical trials for CAR-T cells. In this early stage of clinical development, CAR-T cells offer much hope. The ability of genetic manipulation techniques to modify CAR-T cells provides almost unlimited opportunities for other changes and improvements, thus providing a strong desire for future success [3].

### 3.1. Lack of Proper Targets and Heterogeneity

Having an ideal target is one of the critical reasons why CAR-T cell therapy has achieved impressive results in hematological tumors; however, this is also the most significant obstacle to the CAR-T cell treatment for solid tumors [44]. Compared with hematological tumors, solid tumor tissues are more complex, and the protein expression profiles of different tumor cells vary. It is difficult to select targets that can cover all tumor cells [45]. Importantly, studies have shown that even if antigen-positive tumor cells are cleared, a large number of antigen-negative cells still remain in the body, resulting in an especially high rate of tumor recurrence [46]. Solid tumor cells are derived from healthy tissues. Most antigens that are highly expressed in tumor cells are also expressed in small amounts in healthy cells, so it is challenging to avoid the nonspecific killing of normal cells, which produces on-target, off-tumor side effects [43]. A similar effect can also be observed in CAR-T cell therapy with CD19 as the target antigen. A way to address the safety of CAR-T cell therapy is to reduce the receptor affinity [47]. Another potential solution is to use multitargeted CAR-T cells to reduce the binding of CAR-T cells to the target on healthy tissues [48–50]. Another challenge for CAR-T cell treatment in solid tumors is high tumor heterogeneity, in which tumors in the patient do not all express the same tumor-associated antigen [51]. Therefore, if a single target is recognized CAR-T cells is used to treat a solid tumor, theoretically, the heterogeneity of the solid tumor determines whether the therapy will be successful or fail to eradicate the disease [48, 49]. A feasible way to address tumor heterogeneity is to design multivalent CAR-T cells using genetic engineering techniques. Another necessary method to address tumor heterogeneity is to target cancer stem cells, which are highly correlated with tumor heterogeneity. CD133 is a novel tumor stem cell marker that has been overexpressed in many solid tumors and is now a target tumor marker for CAR-T cells [52]. The high heterogeneity of solid tumors makes their treatment much more difficult than the treatment of hematological malignancies. Therefore, the critical challenge for the treatment of solid tumors with CAR-T cells therapy is whether the appropriate target is selection [53].

### 3.2. CAR-T Cells Are Not Effectively Infiltrating into Tumor Tissue

Because CAR-T cells return to the lymphatic system as well as the blood system, there is a greater chance of contact with blood tumor cells. CAR-T cell therapy for solid tumors is more limited than hematological tumors, depending on whether it can penetrate into the tumor tissue through vascular endothelial cells [54]. Tumor tissue has a mechanism to downregulate the secretion of vascular-related factors. For example, the expression of the endothelin B receptor in tumor tissues is usually high, while the highly expressed endothelin B receptor downregulates the expression of ICAM-1 and inhibits the escape of T cells from blood vessels [55]. After the cells penetrate the vessel wall, they must penetrate the dense tumor tissue to bind to the target cells. It is also worth noting that the migration of CAR-T cells in solid tumors mainly depends on chemokine regulation. However, the expression of these important chemokines in tumors is usually low, such as chemokine ligand-11, factor-10, and chemokine ligand-12 [56, 57].

The low ability of CAR-T cells to migrate and invade tumors is due to the dense fibrotic matrix in solid tumors and downregulate of the expression of chemokines that mediate T cell infiltration into tumor tissues [44, 53]. To achieve effective infiltration of tumor tissue by CAR-T cells, the researchers searched for CAR-T cells that coexpress better-matched chemokine receptors [58]. Oncolytic viruses with chemokines also increase the infiltration capacity of CAR-T cells, which have the ability to specifically infect and lyse tumor cells [59]. Stasi et al. used a subcutaneous lymphoma model to confirm that lymphoma cells expressing the chemokine CCL17 or CCL2 can effectively recruit CAR-T cells expressing CCR4, thereby achieving tumor killing of tumors [60]. Indeed, CAR-T cells expressing high levels of CCR2 receptor can migrate more efficiently to tumor sites secreting CCL2 and have stronger antitumor activity [58, 61]. In addition, the overexpression of HPSE in CAR-T cells can effectively degrade extracellular matrix and effectively infiltrate tumor tissue. Another strategy can be implemented to increase the infiltration of T cells into tumor, to achieve the goal of increasing cells in the tumor that induce local inflammatory conditions using conventional cancer treatment methods such as radiotherapy and chemotherapy [62].

### 3.3. Effect of Hostile Immunosuppressive Tumor Microenvironment on CAR-T Cell Therapy

The tumor microenvironment (TME) is a complex tumor-dependent environment composed mainly of various extracellular matrices (ECMs) and stromal cells, inflammatory cells, and vasculature. Compared with that of hematological tumors, the tumor microenvironment of solid tumors is mostly characterized by low vascularization, hypoxia, and high concentration of extracellular matrix [63]. One of the possible reasons for the unsatisfactory effect of CAR-T cells in solid tumors in vivo is that T cells cannot penetrate these physical barriers and metabolic barriers formed by the TME around tumor tissues recruit immunosuppressive cells [64]. It is worth mentioning that the expression of activators is low in the tumor microenvironment, resulting in inhibition of activation and persistence of engineered T cells [65]. This causes blockade of CAR-T cell migration and immune escape of some tumor cells. Therefore, TME can be modified to improve the therapeutic effect of CAR-T cells in solid tumors [66]. Chemotherapy with cyclophosphamide alone or in combination with fludarabine facilitates T cell implantation and reduces inhibitory immune cells in TME [67].

Currently, clinical trials specifically blocking PD-1 and using CAR-T cell therapy are underway. Tumor cells overexpressing PD-L1 and PD-L2 reduce the tumor suppression mediated by CAR-T cells, thereby increasing tumor cell survival [62]. Targeting immunosuppressive pathways in the TME can cause a sustained antitumor response in advanced-stage patients, with an unusual effect on cancer progression. Studies have shown that specific blockade of PD-1 immunosuppression can effectively enhance CAR-T cell therapy, which is of considerable significance to potentially improving the therapeutic effect of this method in cancer patients [68, 69]. In addition to combining CAR-T cells with checkpoint inhibitors, researchers are also developing alternative therapies that block these inhibitory pathways [70–72].

## 4. Preclinical Studies of CAR-T Cell Immunotherapy

The results of first generation of CAR-T cell therapy in cancer treatment are not satisfactory. However, CAR-T cells targeting the CD19 antigen show good prospects and these CAR-T cells have had intriguing effect in clinical trials [34, 73, 74]. Several key features of CD19 make it a near-ideal therapeutic target: the CD19 antigen is expressed explicitly on the surface of the B cell lineage and B cell malignancies and not on the surface of normal hematopoietic stem cells [75, 76]. The CD19 CAR-T cells of the clinical trial are summarized in Table 2. Clinical studies at several different institutions have used CD19 CAR-T cell therapy in children and adults with recurrent B cell acute lymphoblastic leukemia (B-ALL), B cell non-Hodgkin's lymphoma (B-NHL), and chronic lymphocytic leukemia (CLL), and these studies showed high antitumor treatment efficacy [77, 78]. As of August 2017, there were approximately 200 clinical trials involving CAR-T cells worldwide [79]. In those trials, approximately about 65% of the trials involved hematological malignancies, of which 80% involved CD 19 CAR-T cells targeting B cell cancers [80–82]. Over the past year, more than 200 clinical trials have been conducted worldwide, including trials in liver cancer, breast cancer, neuroblastoma, pancreatic cancer, and glioblastoma [83].

The first-to-market CAR-T cell therapy Kymriah was approved by the FDA in October 2017 [79]. According to the results of clinical trials, Kymriah has a remission rate of 85% after three months when used to treat B cell acute lymphoblastic leukemia (B-ALL). However, 49% of patients develop side effects such as neurotoxicity and cytokine release syndrome, which are one of the causes of death in CAR-T cell therapy [46]. Then, the FDA approved a second CAR-T cell therapy, Yescarta, in October 2010 [70, 79]. Recent reports have proven that Yescarta is secure and controllable over the long term. After two years of follow-up of 101 patients with refractory large B cell lymphoma, a median follow-up of 27.1 months, 39% of patients maintained remission, and 37% of patients maintained complete remission. At two years, the overall survival rate is still 51%. In addition, 93% of patients with remission maintained remission at two years. Data analysis of the 108 patients at two years revealed that there were 12 patients (11%) and 35 patients (32%) with tertiary or higher cytokine syndrome and neurotoxicity, respectively, but these symptoms were overall controllable [28]. Four patients developed serious adverse reactions that were not associated with Yescarta. No new CRS or neurological even or death occurred.

## 5. The Opportunities and Challenges of CAR-T Cells

Although CAR-T cell therapy has been effective in a variety of cancers, there are still some challenges. Currently, to overcome the restrictions of CAR-T cell monotherapy, scientists have attempted to combine CAR-T cells with other therapies to improve the effectiveness of CAR-T cells. Numerous recent reports indicate that the combination of CAR-T cells and chemotherapy can decrease the side effects of the disease, enhance tumor antigen recognition, and increase the efficacy and persistence of CAR-T cells [84–86]. Additionally, studies have shown that the combination of CAR-T cells and radiation therapy can also enhance the efficacy of CAR-T cells. Weiss et al. demonstrated that combined radiotherapy and CAR-T cell therapy could improve the transport and infiltration of T cells, produce synergistic activity, improve tumor antigen presentation, and enhance the durability of CAR-T cells [87]. Some patients will experience antigen escape after receiving CAR-T cell therapy, leading to the failure of CAR-T cells and the recurrence of malignant tumor. The combination of CAR-T cells and checkpoint inhibitor therapy can effectively address this problem and improve this clinical outcome [88]. Current combinations of CAR-T cells and checkpoint inhibitors are effective in mice [88].

Other new attempts include introducing suicide genes or molecular switches, developing multitarget CAR-T cell technologies, resolving potential insertion-related mutations and improving long-term safety by using a nonintegrated lentiviral vector, and generating CAR-T cells with CRISPR/cas9 technology [89]. These gene editing strategies knock out the endogenous TCR and MHC molecules of the transfused CAR-T cells to avoid the host immune rejection and improve the recognition efficiency of CAR-T cells. Current clinical studies have found that CAR-T cells are useful in the treatment of hematological malignancies. In the future, CAR-T cells will become one of the most critical methods in the treatment of hematological malignancies, but their safety and specificity need to be further improved.

## 6. Global Landscape of CAR-T Cell Therapy

In the era of genome-wide date and big data, with the development of technology, more increasing numbers of data are accumulating. CAR-T cells are one of the most critical advances in the field of immunotherapy at the beginning of this century and will probably become an essential immunological technology in the 21st century, ultimately defeat fatal diseases such as cancer.

At present, CAR-T cells are widely used in cellular immunotherapy for various tumors. According to statistics, more than 300 clinical trials of CAR-T cell therapies have been approved by many national drug regulatory agencies, including the FDA of the United States [90, 91]. Statistical data from these clinical trials show that although the effects of various clinical trials vary due to the use of different sources and the preparation techniques of CARs and T cells, as well as differences in pretreatment and combinations of drugs, overall, CAR-T cells are effective in treating tumors with an effective rate of 30% to 70% or even more than 90%. For example, the complete remission rate for r/r ALL treated with the Novartis drug CTL0l9, which the FDA has approved, is 93%. Perhaps CAR-T cell therapy will ultimately remedy the fate of human cancer.

In the development of CAR-T cell therapy, China and the United States are pioneers. Globally, the number of clinical studies of CAR-T cell treatment is increasing dramatically [36]. According to statistics from the Cancer Research Institute (CRI), as of February 2018, a total of 404 CAR-T cell projects worldwide are in clinical trials, mainly led by researchers in China and the United States [92]. Among them, there are 171 clinical trials in the United States and 152 in China. In total, 79.95% of CAR-T cell-based trials worldwide occur in China and the United States, suggesting that these two countries are leading the way for global CAR-T cell immunotherapy [92]. At present, CAR-T cell projects in clinical research involve more than 47 targets. In terms of target distribution, clinical trials of CAR-T cell therapy mainly focus on CD19, CD20, CD22, GPC3, BCMA, and other popular targets. Clinical trials targeting CD19 accounted for more than 40% of the CAR-T cell trials in the United States. Previously, two CAR-T cell products approved by Novartis and Kite Pharma also targeted CD19, and subsequent products have targeted BCMA, CD22, CD30, and other targets [93]. Similar to that in the United States, the number of CAR-T cell clinical trials targeting CD19 in China is also over 40%. However, the second most common target of CAR-T cell clinical trials in the United States is BCMA of CD19, while the number of clinical trials targeting BCMA in China is less than targeting CD20, CD22, and GPC3, accounting for only 5%.

## 7. Conclusions

CAR-T cells have shown amazing promise strength in the treatment of hematological tumors; however, due to lack of a suitable antigen and the hostile immunosuppressive tumor microenvironment, CAR-T cells are not effective in infiltrating into tumor tissue. CAR-T cells also face many difficulties in solid tumors. Future treatment schemes for tumors must be built on the combination of immunotherapy-based treatment with other methods. In the future, adoptive cell therapy, microenvironmental modification, and immune blockade strategies should be combined. CAR-T cells are only the beginning of immunotherapy rather than the end.

## Figures and Tables

**Table 1:** Treatment and challenge of CAR-T cell therapy in the treatment of solid tumors.

Clinical challenges	Strategy	Expected outcome
Lack of specific targets [44]	Designed an antigen-specific inhibitory CAR-T molecule and a dual target CAR-T cells [48, 94]	Achieving dynamic and safe regulation of CAR-T cells [49]
CAR-T cell cannot effectively migrate and infiltrate tumor tissue [44]	Overexpression of HPSE in CAR-T cells can effectively degrade extracellular matrix and effectively infiltrate tumor tissue	Applied to solid tumor treatment [94]
Effect of tumor microenvironment on CAR-T cell therapy [44]	Specific blocking of immune checkpoint inhibition [48, 49, 94]	Improving the therapeutic effect of CAR-T cells in solid tumor [48, 94]
Tumor heterogeneity [52]	Expanding the scope of CAR-T cell therapy targeting tumor cells	Reduce tumor cells to immune response without immune response or low response to immune escape

**Table 2:** CD19 CAR-T of clinical trial.

Study (reference)	Patient number	scFv	Costimulatory domain	Median age (range) (y)	CAR-T cell doses	Disease-related outcomes
Maude et al. (2014) [28]	30 ALL (25 children, 5 adults)	FMC63	4-1BB	Pediatric ALL: 11 (5–22) Adult ALL: 57 (36–60)	0.76‐20.6 × 106 CAR + T cells/kg	6-month EFS: 67% 6-month OS: 78% CR: 90% (MRD-negative in 88% of those who achieved CR)
Brentjens et al. (2011) [81]	8 CLL adults	SJ25C1	CD28	Adults CLL: 58 (67-48)	Cohort receiving no LDC: 1.2–3.0 × 107 CAR + T cells/kg Cy cohort: 0.4‐1.0 × 107 CAR + T cells/kg	ORR: 1/8 (PR); two others with ≥2 months of SD, all in Cy cohort
Brudno et al. (2016) [80]	5 ALL	FMC63	CD28	Adults ALL: 25 (20-68)	5.2‐7.0 × 106 CAR + T cells/kg	CR: 80% (4/5, all MRD-negative)
Kochenderfer et al. (2015) [82]	5 CLL adults	FMC63	CD28	Adults CLL: 61(48-68)	1 × 106 CAR + T cells/kg	CR: 60%
Lee et al. (2014) [76]	20 B-ALL	FMC63	CD28	Children and young adults: 15 (1-30)	1 × 10^6^ CAR-transduced T cells per kg (dose 1) 3 × 10^6^ CAR-transduced T cells per kg (dose 2)	CR: 70% (MRD-negative in 86% of those who achieved CR) PD: 20% & SD: 10% (day 28)
Schuster et al. (2017) [18]	28 lymphomas	FMC63	4-1BB	Adult patients	Median total CTL019-cell: 5.00 × 108 (range, 1.79 × 108 to 5.00 × 108) Median CTL019-cell: 5.79 × 106 cells/kg (range, 3.08 × 106 to 8.87 × 106 cells/kg)	CR at 6 months: (57%; 95% CI, 37 to 76) CR: 57% median of 28.6 months (range, 3.5 to 37.9)

EFS: event-free survival; CLL: chronic lymphocytic leukemia; CR: complete response; PR: partial response; SD: stable disease; PD: progressive disease.

## Abbreviations

CAR-T: Chimeric antigen receptor T
ACT: Adoptive cell transfer therapy
TILs: Tumor-infiltrating lymphocytes
TCR: T cell receptor
MHC: Major histocompatibility complex
ITAM: Immunoreceptor tyrosine-based activation motif
FDA: Food and Drug Administration
TAA: Tumor-associated antigen
B-ALL: B cell acute lymphoblastic leukemia
CLL: Chronic lymphocytic leukemia
B-NHL: B cell non-Hodgkin's lymphoma
CRS: Cytokine release syndrome
CRES: CAR-T-related encephalopathy syndrome
TME: Tumor microenvironment
scFv: Single-chain antibody fragment.
